# Can Isoflurane and Meloxicam Mitigate Pain Associated with Cautery Disbudding of 3-Week-Old Goat Kids?

**DOI:** 10.3390/ani10050878

**Published:** 2020-05-18

**Authors:** Melissa N. Hempstead, Joseph R. Waas, Mairi Stewart, Vanessa M. Cave, Mhairi A. Sutherland

**Affiliations:** 1 AgResearch Ltd., Ruakura Research Centre, Hamilton 3240, New Zealand; vanessa.cave@agresearch.co.nz (V.M.C.); mhairi.sutherland@agresearch.co.nz (M.A.S.); 2 School of Science, The University of Waikato, Hamilton 3216, New Zealand; joseph.waas@waikato.ac.nz; 3 InterAg, Ruakura Research Centre, Hamilton 3240, New Zealand; stewartmairi42@gmail.com

**Keywords:** behaviour, cortisol, disbudding, goat, caprine, pain relief, welfare, small ruminant

## Abstract

**Simple Summary:**

Cautery disbudding is commonly carried out on goat kids less than a week of age to prevent horn growth; however, due to differences in management across farms, older goat kids (up to 3 weeks of age) can be disbudded. We evaluated the effect of pain mitigation strategies (isoflurane and meloxicam) on the behaviour and physiology of 3-week-old cautery-disbudded goat kids. We found weak statistical evidence that cortisol concentrations were lower in goat kids that were administered isoflurane (with or without meloxicam) compared to those disbudded without pain relief. However, no other physiological or behavioural measures were affected by the pain mitigation treatments. Further research is needed to determine whether isoflurane (with or without meloxicam) provides sufficient pain relief for disbudding 3-week-old goat kids.

**Abstract:**

We evaluated the effect of pain mitigation strategies (isoflurane and meloxicam) on the behaviour and physiology of 3-week-old disbudded goat kids. Fifty Saanen does (mean ± SD, 21 ± 3 days old) were randomly allocated to one of five treatments: (1) cautery-disbudded (CAUT), (2) CAUT + isoflurane (ISO), (3) CAUT + isoflurane + meloxicam (ISO + MEL), (4) CAUT + meloxicam (MEL), and (5) handled without disbudding or pain relief (SHAM). Blood samples were taken immediately prior to treatment and at 15-, 60- and 120-min post-treatment to assess cortisol, glucose and lactate concentrations. Behaviour (head shaking and scratching, body shaking, feeding and self-grooming) was observed for 1 h pre- and post-treatment using video-cameras. ISO + MEL and ISO kids had lower cortisol concentrations than CAUT kids 15 min post-treatment (*p* ≤ 0.05). There was no effect of treatment or time for glucose and lactate concentrations (*p* ≥ 0.62). At 35 min post-treatment, CAUT, MEL and ISO kids performed more head shakes than SHAM kids (*p* ≤ 0.05). Isoflurane, with or without meloxicam, may reduce acute stress associated with disbudding of 3-week-old goat kids. More research is needed to assess whether isoflurane (with or without meloxicam) can provide sufficient pain relief for disbudding 3-week-old kids.

## 1. Introduction

Cautery disbudding is performed on goat kids and calves to destroy the horn buds, thus preventing horn growth. Horns can cause injuries to other goats [1] or human handlers, damage farm facilities [2] and increase space requirements at feed racks [3]. Cautery disbudding causes thermal burns and inflammation [4], which are considered painful as goat kids attempt to escape and show increased frequencies of vocalisations and leg shaking during disbudding [5,6,7]. Following disbudding, goat kids also show higher rates of head shaking and scratching, elevations in serum or plasma cortisol concentrations and increased tissue sensitivity compared with handled controls [4,8,9]. Therefore, there is a need to establish and validate effective pain mitigation for disbudded goat kids.

Pain in animals cannot be directly measured, but a variety of indicators of pain can be evaluated to provide a more accurate assessment than any single indicator [10]. Physiological changes, such as increases in cortisol concentrations in response to cautery disbudding, have been shown in calves [11,12,13] and kids [5,9,14]. Cortisol is a stress hormone that becomes elevated in response to stressors including pain and is widely accepted as a useful indicator of pain reviewed by Mellor et al., [15]. Other blood constituents that can indicate pain include lactate and glucose concentrations. Lactate can increase in response to cortisol secretion in pigs due to stress causing the mobilization of glycogen stores [16,17]. Glucose is synthesised by gluconeogenesis that is stimulated by glucocorticoids including cortisol [18]. Changes in behaviour (e.g., vocalizations, struggles) can also provide sensitive indicators of pain as they usually occur during or immediately after a painful procedure [15]. Studies by our group have previously validated the use of behavioural indicators of pain associated with disbudding of goat kids [9,19,20].

Local anaesthetics such as lidocaine are commonly used to reduce or eliminate the behavioural or physiological indicators of pain associated with disbudding of calves [21], but there are inconsistent reports of efficacy in reducing these same indicators of pain in goat kids [5,7,22,23]. General anaesthesia using isoflurane has been shown to safely induce unconsciousness in goats [24,25] and reduce behavioural and physiological indicators of pain associated with cautery disbudding of 4-day-old goat kids [9]. The state of unconsciousness results from intoxication of the central nervous system, and patients neither perceive nor recall stimuli, negative or otherwise [26]. Moreover, isoflurane has the added benefit of reducing struggling during disbudding [27], potentially improving the efficiency of the procedure. Additionally, meloxicam can reduce behavioural indicators of pain in 17-day-old kids for up to 7 h after disbudding [14]. Non-steroidal anti-inflammatory drugs (NSAID), such as meloxicam, block cyclooxygenase activity, which inhibits production of prostaglandins that mediate pain and inflammation [28,29].

It is generally considered best practice to disbud goat kids within the first week of life, before the horn bud fuses with the underlying frontal bone [2,30]; this likely reduces the incidence of scurs, which are partial horn regrowths. Horn buds generally attach to the underlying frontal bone and form horns around 1–2 months of age in goat kids [31], compared with 3–6 months of age for calves [32,33]. This difference explains why kids require earlier disbudding than calves. Cautery disbudding can cause damage to the skull (1/70 kids; [4]) and brain of goat kids (1/243 kids; [34]), which can result in meningoencephalitis and mortality [35,36,37]. Increased risk of damage may be associated with the early age at which goat kids are typically disbudded (5–7 days of age; [2]), and/or the relative thinness of the skull. Anecdotally, some operators prefer to disbud goat kids up to 3 weeks of age as kids are considered more robust at this age and better able to handle the trauma of disbudding. There is conflicting evidence of the effect of age on acute pain in calves. Age may not influence pain or peripheral sensitization in calves disbudded at 1 or 4 weeks of age [38,39,40], but 1.5-month-old calves castrated using a Burdizzo showed an increase in cortisol concentrations compared with 5.5-month-old calves [41]. However, to the authors’ knowledge, there are no studies that have investigated the use of general anaesthesia and NSAID alone or in combination for 3-week-old goat kids; however, an earlier study found that meloxicam reduced pain in 17-day-old kids (for up to 7 h after disbudding [14]). Therefore, the objective of our study was to evaluate the effect of isoflurane and meloxicam, alone or in combination, on the behaviour, physiology and live weight gain of 3-week-old dairy goat kids that were cautery-disbudded. We predicted that isoflurane (with or without meloxicam) would reduce indicators of acute pain in disbudded goat kids and that meloxicam would further reduce these indicators of pain, but to a lesser extent.

## 2. Materials and Methods

### 2.1. Animals and Husbandry

The trial was conducted at the Ruakura Research Farm in Hamilton, New Zealand in July and August 2015 (Austral winter). The study was approved by the Ruakura Animal Ethics Committee (Protocol No. 13589). Fifty Saanen or Saanen cross does, aged between 16 and 26 days (mean ± SD, 21 ± 3 days) with an average weight of 5.8 ± 1.0 kg, were used. Kids were sourced from a private commercial farm in the Waikato region at approximately 3 days of age and were housed at the study facility for the entirety of the study. Kids were enrolled in the study approximately 3 days before data collection began. Kids were selected for inclusion in the study if they had horn buds (i.e., not polled), were in good health and were between 2–4 weeks of age at the study’s onset. At enrolment, kids were weighed, given a collar with identification number and marked with paint to identify each kid during subsequent video analysis (i.e., a line across the shoulders, or along the spine, a double line across the rump, a cross on the rump or left unmarked).

The animals were housed in pre-treatment pens (1.64 × 2.40 × 1.50 m high) in groups of five and remained with the same pen-mates for the entire trial. The concrete pen flooring was covered in untreated pine wood shavings (PGG Wrightsons, Hamilton, New Zealand) approximately 10 cm deep. Pens were numbered/colour-coded to facilitate handling and identification of kids during blood sampling and video analysis. Kids were fed 500 mL of milk replacer (Anlamb, Fonterra Ltd., Auckland, New Zealand) via a 10-space milk feeder (Milk Bar, Waipu, New Zealand) twice daily at approximately 0800 and 1600 h (i.e., 1000 mL/kid/day). As the kids grew, this amount was increased as per milk replacer recommendations. Individual milk intake was not measured. Water was provided ad libitum. 

### 2.2. Experimental Design

A randomized complete block design was used, blocked by pen within treatment day. The kids were randomly allocated to one of five treatments balanced for age (n = 10 kids/treatment). A power analysis was carried out to determine the sample size and was based on a 5% significance level and had 80% power. The primary outcomes used were struggling frequency and plasma cortisol concentrations, which were assumed to be normally distributed. For cortisol concentrations, the minimum response to be detected between treatments was estimated to be 55.0 nmol/L, based on results from Alvarez et al. [7] and with a standard deviation of 39.8 nmol/L. The minimum response to be detected between treatments for struggling frequency (during disbudding) was estimated to be 3.2, based on results from Alvarez et al. [7] and with a standard deviation of 2.3.

The order of treatment was randomly generated using Genstat software (Version 16, VSN International Ltd., Hemel Hempstead, UK). There was only one kid per treatment in each pen, and all kids from the same pen were treated on the same day. Treatments were conducted over four treatment days within a 2-week period, with a minimum of two kids per treatment (i.e., 10 kids) tested per experimental day. The kids were fed approximately 1 h prior to treatment, and then collected from their pre-treatment pen and restrained (device described in Hempstead et al. [9]). Hair covering the horn bud area was removed using electric clippers (Laube, 505 cordless kit, Shoof International Ltd., Cambridge, New Zealand).

Treatments were carried out by the same veterinary surgeon between 0830 and 1030 h. Two handlers transported the kids between the treatment and pen areas and helped to restrain the kids (if required). Treatments were modified from those described previously [9]: (i) kids were disbudded using a cautery iron (CAUT; Quality Electric Debudder, 230 V, 190 W; Lister GmbH, Lüdenscheid, Germany) that was heated for approximately 20 min before use (manufacture specification of 600 °C) and held on each horn bud for 1–3 applications of no more than 8 s in total. The use of downwards pressure (and a circular motion of the cautery iron) cut the skin and, with a flick, removed the horn bud. (ii) Isoflurane (ISO) was administered at a rate of 4% via a face mask (in oxygen); delivery continued until the animal lost consciousness (determined by dilation of pupils and loss of the palpebral reflex), at which point the isoflurane mixing rate was reduced to 2%. Kids were then disbudded using the same procedure as for CAUT. Following disbudding, isoflurane was removed from the gas supply, allowing the inhalation of pure oxygen for 5–15 s. The oxygen was then turned off, the face mask removed, and the animal remained in the restraint under supervision until it regained consciousness (≤5 min). (iii) Isoflurane was administered as above, and meloxicam was injected prior to disbudding (ISO + MEL) following the procedure described for MEL below. (iv) Meloxicam (Loxicom 20 mg/mL solution for injection for cattle, pigs and horses, Norbrook Laboratories Ltd, Newry, UK) was injected s.c. (MEL; 0.5 mg/kg BW) over the ribs immediately prior to disbudding—the kids were then disbudded using the same procedure as for CAUT. Meloxicam was administered at the time of disbudding to minimize animal handling. (v) Sham handling (SHAM) involved applying a cold disbudding iron to the horn buds (i.e. not disbudded) for ≤8 s, with no pain relief administered.

After horn buds were removed, antibacterial spray (Tetravet, Bayer New Zealand Ltd., Auckland, New Zealand) was applied to the open wounds to reduce the risk of infection. Sham-handled kids also received antibacterial spray to prevent observer bias during video analysis (i.e., all animals had blue stains over horn bud positions). The kids were then placed in post-treatment pens (1.64 × 2.40 × 1.50 m high), positioned adjacent to the pre-treatment pens, with the same pen-mates. Post-treatment pens, which were identical to the pre-treatment pens, were used to ease handling of the animals during treatment and blood sampling.

### 2.3. Blood Sampling

Plasma cortisol, glucose and lactate concentrations were measured from 4-mL blood samples collected by venipuncture from either jugular vein immediately prior to treatment (baseline), and at 15, 60 and 120 min following treatment. Blood samples were collected from all animals by trained, experienced technicians using 22 g 2.5 cm needles. One handler restrained the kid on a knee (whilst sitting), as another handler drew the blood sample. Each blood draw took approximately 30 s per kid and was carried out in an area immediately adjacent to the pen of each animal. Samples were collected in fluoride oxalate tubes (Becton Dickinson Vacutainer Systems, Franklin Lakes, NJ, USA). Samples were then centrifuged at 3000 rpm for 10 min (approximately 1500 g) at 4 °C and the plasma was separated and stored at −20 °C until analysed (approximately 2 days).

Samples were analysed by a commercial laboratory using standard quality control methodologies. Plasma cortisol concentrations were determined by electrochemiluminescence immunoassay using a commercial kit (Roche Diagnostics GmbH, Mannheim, Germany). Sensitivity of the assay was 1.5 nmol/L. Plasma glucose concentrations were determined by the hexokinase method using a commercial kit (Roche Diagnostics GmbH, Mannheim, Germany). Sensitivity of the assay was 0.1 nmol/L. Plasma lactate concentrations were determined by enzymatic methods using a commercial kit (Roche Diagnostics GmbH, Mannheim, Germany) and sensitivity of the assay was 0.2 nmol/L.

### 2.4. Rectal Temperature

Rectal temperature was measured immediately following blood sampling at each time point using a Rapid Digital Thermometer (Vet Temp^®^, Advanced Monitors Corp., San Diego, CA, USA).

### 2.5. Body Weight

Body weight measurements were taken before the milk was replenished each morning at 24 h pre- (baseline) and 24 and 48 h post-treatment using a Veterinary Platform Scale (Model WS204; Wedderburn, Hamilton, New Zealand).

### 2.6. Behaviour

Video cameras (HDR-CX220E, Sony Corp., Shanghai, China) were used to record kid behaviour for 24 h pre- and post-treatment. However, only activities 1 h before and after treatment were measured, as major differences between treatments were only detected during this timeframe in an earlier study by our group [9]. The 1-h observation period began for each kid after they were placed in the post-treatment pen. The pre-treatment measures occurred at the same time of day as the post-treatment measures, but were recorded on the previous day (i.e., the day prior to disbudding). Based on the results of our earlier study, which inspected the acute behavioural response following treatment [9], the behavioural observations for each kid were grouped into 12 5-min periods within the 1-h post-treatment period (considered with respect to the average of the 12 5-min periods from the pre-treatment hour). The cameras were placed 1.85m above the pens and were fitted with fisheye lenses (Raynox, Insta-Wide lenses, QC-303, Yoshida Industry Co. Ltd., Tokyo, Japan) to enable a full view of each pen.

The monitored behaviours were chosen from an ethogram previously validated by our group [9,19,20], which included head shaking and scratching, body shaking, feeding and self-grooming (Table 1). The frequency of all behaviours was assessed, in addition to the total durations of head scratching, self-grooming and feeding events.

Two trained observers analysed the video-recordings using Adobe Premier Pro software (CS6, Version 6.0.0, Adobe Systems, San Jose, CA, USA). The duration of each event was generated by the software (for both total-h and 5-min-period data). The observers were blind to the treatment each kid received, as the horn buds of all treatments were sprayed with the same coloured antiseptic spray (including SHAM kids). Frequency and duration measures were recorded against kid identification, detected using the markings on the kids’ backs. Tests of intra-observer reliability were carried out using three kids (randomly chosen) within the same 1-h period for each behaviour (kappa, _Κ_ = 0.80 for head shaking; _Κ_ = 0.87 for head scratching; _Κ_ = 0.84 for self-grooming; _Κ_ = 1.00 for feeding and _Κ_ = 1.00 for body shaking). In addition, inter-observer reliability was determined by randomly selecting a further three kids for each behaviour (_Κ_ = 0.88 for head shaking; _Κ_ = 0.83 for head scratching; _Κ_ = 0.87 for self-grooming; _Κ_ = 0.83 for feeding and _Κ_ = 1.00 for body shaking).

### 2.7. Statistical Analysis

Data were analysed using Genstat software (Version 18, VSN International, Hemel Hempstead, UK). Residual plots for all analyses were assessed to detect departures from the underlying model assumptions of normality, independence and constant variance. Log transformations were required for all of the 5-min period behaviour data to stabilize the variance. Mean cortisol, glucose and lactate concentrations, rectal temperature, body weight and total frequency and duration of behaviour (over 1 h post-treatment) were expressed as a difference from baseline values (i.e., the pre-treatment values were subtracted from the post-treatment values). Data from one kid was excluded from all analyses as it suffered an allergic reaction after receiving its treatment (MEL). Due to a video camera malfunction, behavioural data from five kids were not available.

The changes in cortisol, glucose and lactate concentrations, rectal temperature and body weight data were run through separate repeated measures models fitted by restricted maximum likelihood (REML). The model included the fixed effects for treatment, time, and their interaction, and the random effects for kid and kid within time, age, weight (as a variate), breed and pen within treatment date (i.e., the blocking variable). The correlation structure on the same kid over time was modelled using a power model of order 1, with the allowance for heterogeneity over time.

The changes in behaviour frequency and duration data across pre- and post-treatment hours were analysed using a one-way analysis of variance (blocked by pen within treatment date). Further analyses were carried out on head shaking, head scratching and self-grooming data within the first hour post-treatment (i.e., 12 5-min periods) using a linear mixed model fitted by REML. The model included the fixed effects for treatment, time (5-min periods) and their interaction and the random effects for kid, and kid within time, age, weight (variate), breed and pen within treatment date. The average of the 12 5-min periods for the pre-treatment hour was used as the covariate. Due to low frequencies of feeding and body shaking during the 5-min periods, the data were converted to a binary outcome, where 1 represented an occurrence within the 5-min period and 0 represented no occurrence. The binary response was analysed using a linear mixed model fitted by REML. The fixed and random variables were the same as the head shaking, head scratching and self-grooming data within the first hour post-treatment (described above). The correlation between observations on the same kid over time were modelled using an autoregressive model of order 1. Frequency and duration data were highly correlated (r ≥ 0.75); therefore, only frequency data will be presented.

Fisher’s unprotected least significant differences test was used to detect differences between and within treatments. Mean values (back-transformed if required, with exact 95% confidence intervals) were provided with standard error of the difference and the level of significance was set at *p* ≤ 0.05.

The data used in this study can be found in Table S1 in the supplementary materials .

## 3. Results

### 3.1. Cortisol, Lactate and Glucose Concentrations

**Figure 1:**
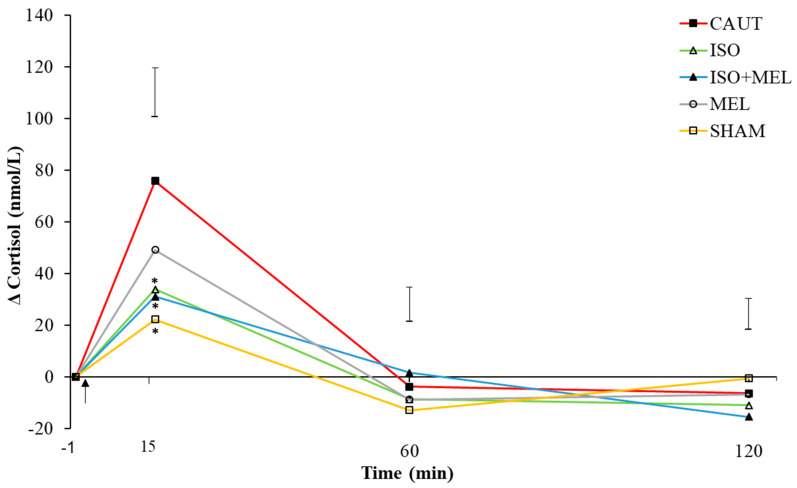
Mean change (± maximum standard error of the difference) in plasma cortisol concentrations (from baseline; nmol/L) over 2 h post-treatment of goat kids (n = 10/treatment) that were either (i) cautery-disbudded with no pain relief (CAUT) or disbudded following administration of (ii) isoflurane (ISO), (iii) meloxicam s.c. and isoflurane (ISO + MEL) or (iv) meloxicam s.c. alone (MEL). (v) Sham-handled kids (SHAM) acted as controls. Asterisks represent means that differ from CAUT kid means within each time point at *p* ≤ 0.05. Arrow indicates time of treatment.

For change in cortisol concentrations, the time effect was highly significant (*F*_2, 45_ = 39.2, *p* < 0.001), but there was no evidence of a treatment effect (*F*_4, 41_ = 0.24, *p* = 0.91). However, there was weak statistical evidence of an interaction of treatment and time (*F*_8, 60_ = 2.0, *p* = 0.07; Figure 1). All kids, regardless of treatment, had increased cortisol concentrations 15 min post-treatment (*p* ≤ 0.05). However, the effect was greater in CAUT than SHAM kids when considering only the first 15 min (*p* ≤ 0.05; Figure 1). The change in cortisol concentration was smaller in ISO and ISO + MEL than CAUT kids 15 min post-treatment (*p* ≤ 0.05; Figure 1). Cortisol concentrations of all kids returned to basal levels by 60 min post-treatment and did not change for the remainder of the study (*p* > 0.05). 

There was no treatment-by-time interaction for change in glucose concentrations (*F*_8, 63_ = 0.8, *p* = 0.62; Table 2). However, there was a time effect (*F*_2, 49_ = 6.6, *p* = 0.003) and weak statistical evidence for a treatment effect (*F*_4, 36_ = 2.4, *p* = 0.07). The changes in glucose concentrations were higher at 120 min post-treatment than the other sampling times (0.37 ± 0.11 mmol/L, −0.02 ± 0.13 mmol/L and 0.05 ± 0.11 mmol/L for 120, 60 and 15 min, respectively; *p* ≤ 0.05). The changes in glucose concentrations were not different between CAUT- and SHAM-treated kids (0.03 ± 0.37 mmol/L and 0.55 ± 0.37 mmol/L; *p* > 0.05). Kids treated with ISO + MEL had a smaller change in glucose concentration than SHAM and MEL kids (−0.53 ± 0.37 mmol/L, 0.55 ± 0.37 mmol/L and 0.40 ± 0.37 mmol/L for ISO + MEL, SHAM and MEL, respectively; *p* ≤ 0.05). 

There was no treatment-by-time interaction for change in lactate concentrations (*F*_8, 62_ = 0.7, *p* = 0.69; Table 2). In addition, there were no effects of treatment (*F*_4, 41_ = 2.0, *p* = 0.11) or time (*F*_2, 47_ = 0.5, *p* = 0.59). 

Treatment-by-time interaction ^a^ *p* = 0.62; ^b^ *p* = 0.68.

### 3.2. Rectal Temperature

**Figure 2:**
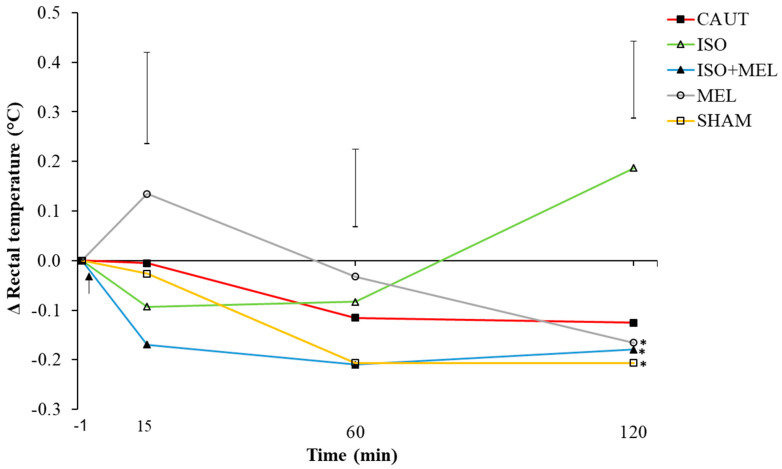
Mean change (±maximum standard error of the difference) in rectal temperature (from baseline; °C) over 2 h post-treatment of goat kids (n = 10/treatment) that were either (i) cautery-disbudded with no pain relief (CAUT), or disbudded following administration of (ii) isoflurane (ISO), (iii) meloxicam s.c. and isoflurane (ISO + MEL) or (iv) meloxicam s.c. alone (MEL). (v) Sham-handled kids (SHAM) acted as controls. Asterisks represent means that differ from ISO kid means within each time point at *p* ≤ 0.05. Arrow indicates time of treatment.

There was a treatment-by-time interaction for change in rectal temperature (*F*_8, 65_ = 2.2, *p* = 0.04; Figure 2). At 120 min post-treatment, ISO kids had a greater change in rectal temperature than kids experiencing other treatments (except for CAUT kids) (*p* ≤ 0.05). At 15 and 60 min post-treatment, the change in rectal temperature did not differ across treatments (*p* > 0.05). There were no differences in the change in rectal temperature between CAUT and SHAM kids post-treatment (*p* > 0.05).

### 3.3. Body Weight

There was no treatment-by-time interaction for mean weight gain (*F*_4, 44_ = 1.6, *p* = 0.19) or a treatment effect (*F*_4, 33_ = 0.5, *p* = 0.73). However, there was a time effect (*F*_2, 48_ = 14.5, *p* < 0.001). Over the two days following treatment, there was increased weight gain from 0.6 to 0.8 ± 0.05 kg (*p* ≤ 0.05). 

### 3.4. Behaviour

#### 3.4.1. 1-h Period

There was no treatment effect on the change in head shaking frequency (F_4, 31_ = 1.6, *p* = 0.20), body shaking frequency (F_4, 31_ = 1.2, *p* = 0.32), head scratching frequency (F_4, 31_ = 0.6, *p* = 0.66), or self-grooming frequency (F_4, 31_ = 0.9, *p* = 0.46) 1 h post-treatment (Table 3).

There was a treatment effect on the change in feeding frequency 1 h post-treatment (F_4, 31_ = 3.0, *p* = 0.04; Table 3). Kids that were treated with MEL fed more often than SHAM and ISO kids (Table 3; *p* ≤ 0.05). There was no difference in feeding frequency between kids treated with CAUT and ISO + MEL or between those treated with ISO and SHAM (Table 3; *p* > 0.05).

#### 3.4.2. 5-min Periods

There was a treatment-by-time interaction for head shaking frequency (*F*_44, 453_ = 1.4, *p* = 0.04). At 35 min post-treatment, SHAM kids performed less head shaking (0.4 [−0.05, 1.18] no./5 min) than CAUT, ISO and MEL kids (2.4 [1.24, 4.11], 1.7 [0.76, 3.02] and 1.75 [0.82, 3.15] no./5 min, respectively; *p* ≤ 0.05).

There was no treatment-by-time interaction (*F*_44, 451_ = 1.2, *p* = 0.16) or treatment effect for body shaking frequency (*F*_4, 31_ = 1.2, *p* = 0.32), although there was a time effect (*F*_11, 460_ = 5.7, *p* < 0.001): there were three times as many body shakes 5 min post-treatment than 55 min post-treatment (*p* ≤ 0.05).

There was no treatment-by-time interaction for head scratching frequency (*F*_44, 453_ = 0.7, *p* = 0.93) or a treatment effect (*F*_4, 35_ = 1.1, *p* = 0.39). However, there was a time effect (*F*_11, 467_ = 6.1, *p* < 0.001). Head scratching was five times as frequent 30 min post-treatment than 5 min post-treatment (*p* ≤ 0.05).

There was no treatment-by-time interaction for self-grooming frequency (*F*_44, 453_ = 0.6, *p* = 0.99) or a treatment effect (*F*_4, 34_ = 0.2, *p* = 0.93). However, there was a time effect (*F*_11, 467_ = 5.3, *p* < 0.001). All kids performed six times as many self-grooming events 20 min post-treatment compared with those 5 min post-treatment (*p* ≤ 0.05).

There was no treatment-by-time interaction for feeding frequency (*F*_44, 451_ = 0.6, *p* = 0.98) or a treatment effect (*F*_4, 34_ = 1.5, *p* = 0.22). However, there was a time effect (*F*_11, 423_ = 8.6, *p* < 0.001). Feeding was five times as frequent 5 min post-treatment compared to 60 min post-treatment (*p* ≤ 0.05).

## 4. Discussion

We examined the effect of isoflurane and meloxicam, alone or in combination, on the behaviour, physiology and live weight gain of 3-week-old cautery-disbudded goat kids. Although there was no clear treatment effect, there was weak statistical evidence for lower cortisol concentrations of kids disbudded while anesthetized with isoflurane compared with those that were disbudded only; this indicates that isoflurane, to some degree, may have reduced the acute stress response associated with disbudding. In previous research, 4-day-old goat kids that were disbudded whilst under isoflurane (with or without meloxicam) had lower cortisol concentrations and performed fewer head and body shakes than kids disbudded without pain relief, during the first hour post-treatment [9]. However, it is possible that isoflurane itself altered the cortisol concentrations of the kids that were administered anaesthesia during disbudding. Calves that were administered isoflurane during umbilical surgery displayed an increase in cortisol until 15 min into the operation, which decreased from then on [42]. To examine the effect of isoflurane on the acute stress response of goat kids, an additional treatment group administered isoflurane, but not disbudded, should ideally have been included. Logistic constraints prevented us from including this additional treatment in the study. Isoflurane has been shown to induce and maintain anaesthesia in goats with minimal complications [24,25,43]. Additionally, isoflurane has multiple benefits over other inhalant anaesthetic agents or injectable general anaesthesia as it is minimally metabolised (therefore, the anaesthetic depth can be quickly adjusted), and recovery is faster (which may reduce risks associated with prolonged recumbency; e.g., bloat, reflux, aspiration) [26]. Based on research to date, it appears that isoflurane can reduce the behavioural or physiological indicators of acute pain associated with cautery disbudding of goat kids [9]. However, this method may not be feasible for on-farm use due to the requirement of veterinary administration and specialist equipment, and therefore, other more practical options (e.g., effective local anaesthetic techniques) for reducing or eliminating pain at the time of disbudding should be investigated.

In the present study, there was no evidence to suggest that meloxicam administered immediately prior to disbudding reduced the behavioural or physiological indicators of pain in 3-week-old kids during the first hour post-treatment; behavioural responses were not assessed beyond this time. An earlier study by our group monitored the behaviour of 4-day-old kids over 24 h post-disbudding and found those that were administered meloxicam performed less head scratching than kids that were disbudded without meloxicam 1 h post-treatment [9]. Additionally, the kids that were administered meloxicam had similar head scratching durations as sham-handled kids over the remaining 24 h [9]. Ingvast-Larsson et al. [14] administered meloxicam shortly after disbudding and reported fewer behavioural signs of pain in kids given meloxicam than those disbudded without meloxicam for up to 7 h post-treatment. The observation period may not have been long enough to detect any behavioural differences across treatments in the present study. In our study, meloxicam was administered immediately prior to disbudding for practical reasons. It would be of interest to determine if administering an NSAID at least 20 min prior to disbudding would improve its efficacy at reducing acute pain associated with this procedure.

Goat kids that were disbudded under isoflurane showed no difference in body temperature before or after the procedure, or with other groups that did not receive anaesthesia [9]. Generally, anaesthesia alters thermoregulation, resulting in heat loss and a decrease in body temperature [44]. However, in the present study, kids treated with ISO had statistically higher rectal temperatures 2 h post-treatment when compared to other treatments, with the exception of CAUT; we cannot offer an explanation for this result. Further research may be required to ascertain the effect general anaesthesia has on body temperature of disbudded goat kids.

Activation of the HPA axis causes an elevation of cortisol, which results in increased production of glucose and lactate via glycolysis [26]. In the present study, cortisol concentrations were higher in cautery-disbudded kids than sham-handled kids post-treatment, but glucose and lactate concentrations did not differ between treatments. However, kids that were provided both isoflurane and meloxicam had lower glucose concentrations after treatment than sham-handled kids or disbudded kids administered either isoflurane or meloxicam alone. As the kids that were disbudded without pain relief showed similar glucose concentrations to sham-handled kids, the result may be associated with the physiological consequences of combining meloxicam and isoflurane, rather than pain associated with disbudding [25]. Previous studies also found no difference in glucose concentrations of kids before and after disbudding [9,14]. It appears that neither glucose nor lactate correlate with acute stress or pain associated with disbudding of goat kids.

Meloxicam may have affected feeding rates, as kids administered meloxicam prior to disbudding showed higher feeding frequencies than sham-handled kids and those administered isoflurane. A tendency for meloxicam-treated disbudded calves to feed more often than those not provided meloxicam has been reported previously [45]. Theurer et al. [46] found that calves given meloxicam prior to disbudding spent more time than control calves (not given meloxicam) around the feed bunk. In addition, Todd et al. [47] found that calves treated with meloxicam for neonatal diarrhoea displayed a reduced latency to feed and also ingested higher amounts of feed compared with those not treated with meloxicam. Collectively, these findings may reflect pain alleviation associated with meloxicam, resulting in an increase in appetite. 

Body weight increased steadily for all kids, regardless of treatment. In earlier studies, kids aged between 4 and 11 days that were disbudded (i.e., using either caustic paste, liquid nitrogen, clove oil or a cautery iron), had no difference in average daily gains over 2 weeks in comparison to those that were sham-handled [4,8]. It appears that pain associated with disbudding may not affect the growth rates of goat kids over these timeframes.

To our knowledge, we present the first evidence that isoflurane (with or without meloxicam) may reduce the acute stress response in 3-week-old goat kids; however, the study is not without limitations. To fully comprehend how differences in disbudding age can influence the responses, and the ontogeny of acute pain responses, a study simultaneously comparing 3-week-old kids with younger animals (e.g., ≤1-week-old) would be required.

## 5. Conclusions

There was weak statistical evidence that isoflurane, with or without meloxicam, initially reduced plasma cortisol concentrations in cautery-disbudded 3-week-old goat kids compared with kids disbudded without pain mitigation strategies, and therefore may reduce acute stress associated with this procedure. However, as isoflurane did not appear to affect any other physiological or behavioural responses to disbudding, more research is needed to assess whether isoflurane (with or without meloxicam) provides sufficient pain relief when disbudding 3-week-old goat kids. Although meloxicam used singly appeared to have no effect on reducing the behavioural or physiological indicators of pain within 1 h of disbudding, it did appear to have a positive effect on feeding rates. 

## Figures and Tables

**Table 1:** Ethogram of behaviours quantified in the present study. Modified from Hempstead et al. [9].

Behaviour	Description
Head shaking	Rapid continuous tilting of the head from side to side concluding with a return to neutral position. Head shakes separated by >1 s were considered separate events.
Head scratching	The rear foot touches any part of the head or neck (including collar). Scratches separated by >1 s were considered separate events.
Body shaking	Hackles on the back are raised and body shakes from side to side concluding at a return to neutral position. Body shakes separated by >1 s were considered separate events.
Self-grooming	The kid’s muzzle contacts any part of the body or legs (excluding hoof) with a rhythmic back and forth motion. A separate grooming event was considered to occur after a pause of >1 s.
Feeding	The mouth covers at least half of the nipple of the feeding bucket for >3 s, usually followed by suckling motions. Repetitions following separation of the mouth from the nipple of >3 s were considered separate events.

**Table 2:** Mean change (±maximum standard error of the difference; SED) in plasma glucose and lactate concentrations (from baseline; mmol/L) over 2 h post-treatment of goat kids (n = 10/treatment) that were either (i) cautery-disbudded with no pain relief (CAUT), or disbudded following administration of (ii) isoflurane (ISO), (iii) meloxicam s.c. and isoflurane (ISO + MEL) or (iv) meloxicam s.c. alone (MEL). (v) Sham-handled kids (SHAM) acted as controls.

Plasma Concentrations	Time (min)		
	15	60	120
^a^ Δ Glucose (mmol/L)			
CAUT	<−0.1	<−0.1	0.2
ISO	<0.1	0.2	0.5
ISO + MEL	−0.8	−0.7	−0.1
MEL	0.5	0.2	0.5
SHAM	0.6	0.3	0.8
Max SED	0.4	0.5	0.4
^b^ Δ Lactate (mmol/L)			
CAUT	0.2	<−0.1	−0.1
ISO	−0.2	0.1	−0.2
ISO + MEL	−0.3	−0.2	−0.2
MEL	0.4	0.1	0.3
SHAM	−0.1	−0.6	−0.5
Max SED	0.4	0.4	0.3

Treatment-by-time interaction ^a^ *p* = 0.62; ^b^ *p* = 0.68.

**Table 3:** Mean frequency (no./h) of head shaking, body shaking, head scratching, self−grooming and feeding over 1 h post−treatment (± standard error of the difference). Goat kids (n = 10/treatment) were either (i) cautery-disbudded with no pain relief (CAUT), or disbudded following administration of (ii) isoflurane (ISO), (iii) meloxicam s.c. and isoflurane (ISO + MEL) or (iv) meloxicam s.c. alone (MEL). (v) Sham-handled kids (SHAM) acted as controls. Means with differing superscripts were significantly different at *p* ≤ 0.05.

Behaviours	CAUT	ISO	ISO + MEL	MEL	SHAM	SED
Head shaking (No./h)	24.4	12.6	15.7	20.5	18.0	5.1
Body shaking (No./h)	1.9	1.1	2.9	1.6	2.7	0.9
Head scratching (No./h)	10.6	9.8	7.1	7.0	4.9	4.5
Self-grooming (No./h)	2.9	−0.8	3.0	4.1	5.2	3.3
Feeding (No./h)	4.0 ^a,b^	−1.7 ^a^	5.8 ^a,b^	9.9 ^b^	0.7^a^	3.7

## Supplementary Materials

The following are available online at https://www.mdpi.com/2076-2615/10/5/878/s1, Table S1: Data set used in the present study.

## References

[1] Waiblinger S., Schmied-Wagner C., Mersmann D., Nordmann E. Social behaviour and injuries in horned and hornless dairy goats. Proceedings of the XVth International Congress of the International Society for Animal Hygiene.

[2] Smith M.C., Sherman D.M. (2009). Dehorning and descenting. Goat Medicine.

[3] Loretz C., Wechsler B., Hauser R., Rusch P. (2004). A comparison of space requirements of horned and hornless goats at the feed barrier and in the lying area. Appl. Anim. Behav. Sci..

[4] Hempstead M.N., Waas J.R., Stewart M., Zobel G., Cave V.M., Julian A.F., Sutherland M.A. (2018). Pain sensitivity and injury associated with three methods of disbudding goat kids: Cautery, cryosurgical and caustic paste. Vet. J..

[5] Alvarez L., De Luna J.B., Gamboa D., Reyes M., Sánchez A., Terrazas A., Rojas S., Galindo F. (2015). Cortisol and pain-related behavior in disbudded goat kids with and without cornual nerve block. Physiol. Behav..

[6] Alvarez L., Gutierrez J. (2010). A first description of the physiological and behavioural responses to disbudding in goat kids. Anim. Welf..

[7] Alvarez L., Nava R.A., Ramirez A., Ramirez E., Gutierrez J. (2009). Physiological and behavioural alterations in disbudded goat kids with and without local anaesthesia. Appl. Anim. Behav. Sci..

[8] Hempstead M.N., Waas J.R., Stewart M., Cave V.M., Sutherland M.A. (2018). Evaluation of alternatives to cautery disbudding of dairy goat kids using physiological measures of immediate and longer-term pain. J. Dairy Sci..

[9] Hempstead M.N., Waas J.R., Stewart M., Dowling S.K., Cave V.M., Lowe G.L., Sutherland M.A. (2018). Effect of isoflurane alone or in combination with meloxicam on the behavior and physiology of goat kids following cautery disbudding. J. Dairy Sci..

[10] Broom D.M. (1988). The scientific assessment of animal welfare. Appl. Anim. Behav. Sci..

[11] Allen K.A., Coetzee J.F., Edwards-Callaway L.N., Glynn H., Dockweiler J., KuKanich B., Lin H., Wang C., Fraccaro E., Jones M. (2013). The effect of timing of oral meloxicam administration on physiological responses in calves after cautery dehorning with local anesthesia. J. Dairy Sci..

[12] Faulkner P.M., Weary D.M. (2000). Reducing pain after dehorning in dairy calves. J. Dairy Sci..

[13] Graf B., Senn M. (1999). Behavioural and physiological responses of calves to dehorning by heat cauterization with or without local anaesthesia. Appl. Anim. Behav. Sci..

[14] Ingvast-Larsson C., Hogberg M., Mengistu U., Olsen L., Bondesson U., Olsson K. (2011). Pharmacokinetics of meloxicam in adult goats and its analgesic effect in disbudded kids. J. Vet. Pharmacol. Ther..

[15] Mellor D.J., Cook C.J., Stafford K.J., Moberg G.P., Mench J.A. (2000). Quantifying some responses to pain as a stressor. The Biology of Animal Stress.

[16] Brown S.N., Warriss P.D., Nute G.R., Edwards J.E., Knowles T.G. (1998). Meat quality in pigs subjected to minimal preslaughter stress. Meat Sci..

[17] Hambrecht E., Eissen J.J., Nooijen R.I.J., Ducro B.J., Smits C.H.M., Den Hartog L.A., Verstegen M.W.A. (2004). Preslaughter stress and muscle energy largely determine pork quality at two commercial processing plants. J. Anim. Sci..

[18] Khani S., Tayek J.A. (2001). Cortisol increases gluconeogenesis in humans: Its role in the metabolic syndrome. Clin. Sci..

[19] Hempstead M.N., Waas J.R., Stewart M., Cave V.M., Sutherland M.A. (2017). Behavioural response of dairy goat kids to cautery disbudding. Appl. Anim. Behav. Sci..

[20] Hempstead M.N., Waas J.R., Stewart M., Cave V.M., Sutherland M.A. (2018). Evaluation of alternatives to cautery disbudding of dairy goat kids using behavioural measures of post-treatment pain. Appl. Anim. Behav. Sci..

[21] Stock M.L., Baldridge S.L., Griffin D., Coetzee J.F. (2013). Bovine dehorning: Assessing pain and providing analgesic management. Vet. Clin. N. Am. Food Anim. Pract..

[22] Ajuda I., Battini M., Mattiello S., Arcuri C., Stilwell G. (2020). Evaluation of Pain Mitigation Strategies in Goat Kids after Cautery Disbudding. Animals.

[23] Nfor O.N., Chan J.P.W., Kere M., Peh H.C. (2016). Disbudding pain: The benefits of disbudding goat kids with dexmedetomidine hydrochloride. Small Rumin. Res..

[24] Dzikiti T.B., Stegmann G.F., Dzikiti L.N., Hellebrekers L.J. (2011). Effects of midazolam on isoflurane minimum alveolar concentration in goats. Small Rumin. Res..

[25] McEwen M.M., Gleed R.D., Ludders J.W., Stokol T., Del Piero F., Erb H.N. (2000). Hepatic effects of halothane and isoflurane anesthesia in goats. J. Am. Vet. Med. Assoc..

[26] Dugdale A. (2011). Veterinary Anaesthesia: Principles to Practice.

[27] Riebold T.W., Grimm K.A., Lamont L.A., Tranquilli W.J., Greene S.A., Robertson S.A. (2015). Ruminants. Veterinary Anesthesia and Analgesia: The Fifth Edition of Lumb and Jones.

[28] Dahl J.B., Kehlet H. (1991). Non-steroidal anti-inflammatory drugs: Rationale for use in severe postoperative pain. Br. J. Anaesth..

[29] Del Tacca M., Colucci R., Fornai M., Blandizzi C. (2002). Efficacy and tolerability of meloxicam, a COX-2 preferential nonsteroidal anti-inflammatory drug—A review. Clin. Drug Investig..

[30] Dove W.F. (1935). The physiology of horn growth: A study of the morphogenesis, the interaction of tissues, and the evolutionary processes of a mendelian recessive character by means of transplantation of tissues. J. Exp. Zool..

[31] Hull B.L. (1995). Dehorning the adult goat. Vet. Clin. N. Am. Food Anim. Pract..

[32] McMeekan C.M., Stafford K.J., Mellor D.J., Bruce R.A., Ward R.N., Gregory N.G. (1998). Effects of regional analgesia and/or a non-steroidal anti-inflammatory analgesic on the acute cortisol response to dehorning in calves. Res. Vet. Sci..

[33] Sylvester S.P., Mellor D.J., Stafford K.J., Bruce R.A., Ward R.N. (1998). Acute cortisol responses of calves to scoop dehorning using local anaesthesia and/or cautery of the wound. Aust. Vet. J..

[34] Hempstead M.N., Waas J.R., Stewart M., Cave V.M., Turner A.R., Sutherland M.A. (2018). The effectiveness of clove oil and two different cautery disbudding methods on preventing horn growth in dairy goat kids. PLoS ONE.

[35] Sanford S.E. (1989). Meningoencephalitis caused by thermal disbudding in goat kids. Can. Vet. J..

[36] Thompson K.G., Bateman R.S., Morris P.J. (2005). Cerebral infarction and meningoencephalitis following hot-iron disbudding of goat kids. N. Zea. Vet. J..

[37] Wright H.J., Adams D.S., Trigo F.J. (1983). Meningoencephalitis after hot-iron disbudding of goat kids. Vet. Med. Small Anim. Clin..

[38] Caray D., de Boyer des Roches A., Frouja S., Andanson S., Veissier I. (2015). Hot-iron disbudding: Stress responses and behavior of 1- and 4-week-old calves receiving anti-inflammatory analgesia without or with sedation using xylazine. Livest. Sci..

[39] Mirra A., Spadavecchia C., Bruckmaier R., Gutzwiller A., Casoni D. (2018). Acute pain and peripheral sensitization following cautery disbudding in 1- and 4-week-old calves. Physiol. Behav..

[40] Casoni D., Mirra A., Suter M.R., Gutzwiller A., Spadavecchia C. (2019). Can disbudding of calves (one versus four weeks of age) induce chronic pain?. Physiol. Behav..

[41] Ting S.T.L., Earley B., Veissier I., Gupta S., Crowe M.A. (2005). Effects of age of Holstein-Friesian calves on plasma cortisol, acute-phase proteins, immunological function, scrotal measurements and growth in response to Burdizzo castration. Anim. Sci..

[42] Offinger J., Meyer H., Fischer J., Kastner S.B., Piechotta M., Rehage J. (2012). Comparison of isoflurane inhalation anaesthesia, injection anaesthesia and high volume caudal epidural anaesthesia for umbilical surgery in calves; metabolic, endocrine and cardiopulmonary effects. Vet. Anaesth. Analg..

[43] Antognini J.F., Eisele P.H. (1993). Anesthetic potency and cardiopulmonary effects of enflurane, halothane, and isoflurane in goats. Lab. Anim. Sci..

[44] Lin H.-C., Grimm K.A., Lamont L.A., Tranquilli W.J., Greene S.A., Robertson S.A. (2015). Comparative anesthesia and analgesia of ruminants and swine. Veterinary Anesthesia and Analgesia: The Fifth Edition of Lumb and Jones.

[45] Heinrich A., Duffield T.F., Lissemore K.D., Millman S.T. (2010). The effect of meloxicam on behavior and pain sensitivity of dairy calves following cautery dehorning with a local anesthetic. J. Dairy Sci..

[46] Theurer M.E., White B.J., Coetzee J.F., Edwards L.N., Mosher R.A., Cull C.A. (2012). Assessment of behavioral changes associated with oral meloxicam administration at time of dehorning in calves using a remote triangulation device and accelerometers. BMC Vet. Res..

[47] Todd C.G., Millman S.T., McKnight D.R., Duffield T.F., Leslie K.E. (2010). Nonsteroidal anti-inflammatory drug therapy for neonatal calf diarrhea complex: Effects on calf performance. J. Anim. Sci..

